# ﻿New insights into the *Syllis prolifera* species complex from the eastern Mediterranean Sea

**DOI:** 10.3897/zookeys.1264.170411

**Published:** 2025-12-16

**Authors:** Fernando Belvis, M. Rosario Martín-Hervás, Irene del Olmo, Víctor Ruiz, Patricia Álvarez-Campos

**Affiliations:** 1 Centro de Investigación en Biodiversidad y Cambio Global (CIBC-UAM) & Departamento de Biología (Zoología), Facultad de Ciencias, Universidad Autónoma de Madrid, Madrid, Spain Universidad Autónoma de Madrid Madrid Spain

**Keywords:** Cryptic speciation, integrative taxonomy, species complex, species delimitation, *
Syllis
*

## Abstract

Reports of cases of cryptic and pseudocryptic speciation have increased in the last decade among marine invertebrates thanks to the use of integrative taxonomy that combines all available data sources to correctly establish species boundaries, now routinely incorporated into classical taxonomical studies. This approach has enhanced our understanding of the real biodiversity of a given area, which has fundamental implications for conservation and management of marine ecosystems. Among polychaete annelids, considered one of the most ubiquitous and abundant groups in our oceans, there are numerous documented cases of cryptic and pseudocryptic speciation, mainly in those highly diverse families as Syllidae Grube, 1850. One of the most recent cases of species complex has been reported in *Syllis prolifera* Krohn, 1852 – traditionally considered a widespread, cosmopolitan species – and now recognised as a pseudocryptic complex in the western Mediterranean Sea. However, its broader distribution suggests that additional pseudocryptic lineages may exist in other regions of the Mediterranean basin. In this study, newly collected specimens from the Greek islands of Crete and Mykonos are combined with all previously published data on the *S. prolifera* complex, aiming to assess the existence of additional putative Mediterranean species. Combining morphological, biogeographical, and molecular data (*COI*, *16S rRNA*, *18S rRNA*, and *28S rRNA* markers) the presence of at least three more novel lineages for the eastern Mediterranean Sea is revealed. However, unlike previous studies, no clear diagnostic morphological characters were found for each lineage, suggesting that the complex includes both pseudocryptic and cryptic potential new species. Our results further reinforce the view that species diversity within the family Syllidae remains underestimated and highlight the necessity of integrative studies to accurately assess marine invertebrate biodiversity.

## ﻿Introduction

The Mediterranean Sea harbours a great biodiversity, with approximately 17,000 described species and a high percentage of endemism (Coll et al. 2010), making it an area of high interest for the study of marine ecosystems. However, nearly all regions of the Mediterranean basin face threats such as habitat degradation, overexploitation, pollution, and the spread of invasive species, which contribute to biodiversity loss (Cramer et al. 2020). Furthermore, the underestimation of marine species richness poses a challenge in the context of the global biodiversity crisis (Nygren 2014), highlighting the need to implement conservation strategies based on a deep understanding of the most diverse areas, such as the Mediterranean Sea. Among all the species described in this area, nearly 8,000 are macroscopic invertebrates, including crustaceans, molluscs, and annelids (Musco and Giangrande 2005; Coll et al. 2010). Within the latter, polychaete worms represent one of the most diverse communities in Mediterranean benthic fauna and, specifically in the Aegean Sea, with more than 750 recorded species (Chintiroglou et al. 2005; Çinar et al. 2014, 2024). The family Syllidae Grube, 1850 (Annelida: Phyllodocida) usually stands out among polychaetes due to its great diversity and abundance predominantly in coastal areas, and specifically in the eastern Mediterranean Sea, with more than 100 species reported (Çinar and Ergen 2002; Çinar et al. 2003, 2014, 2024; Rousou et al. 2023; Toso et al. 2024). However, the number of recognised species within this group is continually changing, driven by the exploration of previously unsampled areas (Langeneck et al. 2019) and the application of integrative taxonomy–an approach that has uncovered numerous cases of cryptic and pseudocryptic speciation in syllids (Álvarez-Campos et al. 2017a, 2017b, 2025; Langeneck et al. 2020; del Olmo et al. 2024). Within the diverse type genus *Syllis* Lamarck, 1818 the occurrence of pseudocryptic species complexes was suggested in multiple lineages in nominal taxa with an alleged cosmopolitan distribution (e.g. Aguado et al. 2012; Ba Akdah et al. 2018) and they were studied in detail in two of them, *S. gracilis* Grube, 1840 (Alvarez-Campos et al. 2017a) and *S. prolifera* Krohn, 1852 (del Olmo et al. 2024). Notably, in this latter species, multiple Mediterranean lineages exhibit morphological and ecological distinctions that correspond with substantial molecular divergences (del Olmo et al. 2024). Nevertheless, its widespread distribution suggests that additional pseudocryptic lineages may exist in other regions of the Mediterranean basin, and, therefore, including further localities would be essential for establishing the correct species boundaries within this complex. Thus, we combined the information of newly collected specimens from the Greek islands of Crete and Mykonos with all previously published data on the *S. prolifera* complex to evaluate the potential presence of additional putative species. To test the species status in this region, we combined molecular evidence – using coalescence-based species delimitation methods – with traditional morphological studies. Our work is based on a multilocus dataset comprising two mitochondrial and two nuclear markers, including 31 specimens of *S. prolifera* collected from four localities in the Aegean Sea, as well as 52 additional specimens from other Mediterranean regions.

## ﻿Materials and methods

### ﻿Sampling and morphological study

**Figure 1.**
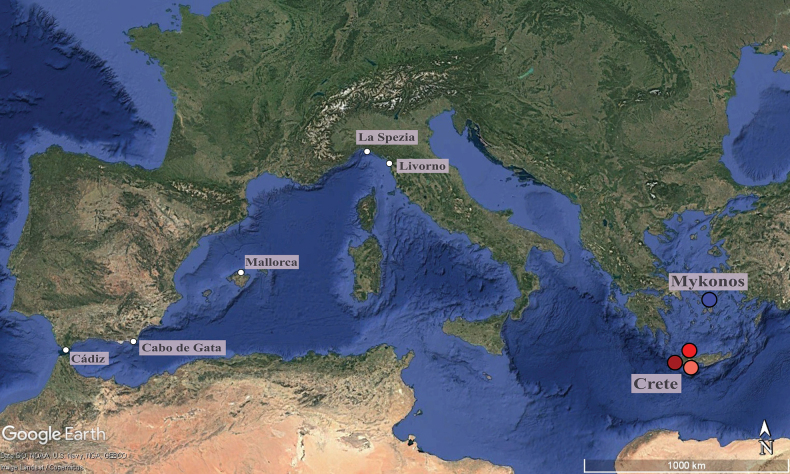
Geographic distribution of the sampling localities in the Mediterranean Sea. Map showing the localities sampled in this study (coloured dots) and other Mediterranean localities of specimens included in the analyses (uncoloured dots) from a previous study (del Olmo et al. 2024). Image source: Google Earth.

Specimens were collected in a scientific survey in July 2024 by hand and snorkelling among algae at four different beaches located on the Greek islands of Crete and Mykonos (Fig. 1). Specimens were sorted and fixed in 96% ethanol and stored at 4 °C in the Department of Biology (Zoology) of the Universidad Autónoma de Madrid (UAM). Further morphological examination and identification was carried out using an Olympus SZ 30 stereomicroscope and an Olympus CX 43 light microscope. Images of the mid and posterior compound chaetae were taken for each morphotype using an Olympus CX 31 microscope equipped with an Olympus SC 50 camera. For scanning electron microscopy (SEM), selected individuals were critical-point dried using an Emitech K850 device, coated with 15 nm of gold in a Q150T-S Turbo-Pumper and then examined with a Hitachi S-3000N at the Servicio Interdepartamental de Investigación (SIDI) of the UAM. Additionally, unpublished SEM images of posterior compound chaetae from individuals analysed in a previous study (del Olmo et al. 2024) were also included to facilitate morphological comparisons with *S. prolifera* specimens from all the Mediterranean localities analysed in the present study. All light microscopy and SEM images were edited in Adobe Photoshop CS6. All specimens used for this study were deposited at the Museo Nacional de Ciencias Naturales de Madrid (MNCN). Voucher numbers, collection date, locality, and other relevant information are listed in Table 1.

**Table 1.** Specimen collection data. Examined specimens with collection localities, substrates, coordinates, catalogue numbers, and GenBank accession numbers for all specimens sequenced. Phylogenetic lineages defined in this study are indicated in bold. Specimens not assigned to any lineage are marked as Not assigned. Dashes (–) indicate absence of data.

Code	Lineage	Locality	Substrate	Coordinates (decimal degrees)	Voucher numbers	*16S*	*COI*	*18S*	*28S*
ELA 1.4	**Lineage 1**	Elafonisi Beach, Crete, Greece	*Corallina* sp. and unidentified green algae	35.271162, 23.541309	MNCN 19484	−	−	PV475185	PV422772
MYK 1.4		Megali Ammos Beach, Mykonos, Greece	*Corallina* sp. and unidentified green algae	37.438991, 25.326656	MNCN 19485	−	PV422749	PV475194	PV422776
MYK 1.2				MNCN 19486	−	PV422753	PV475189	PV422779
MYK 1.1	**Lineage 2**	Megali Ammos Beach, Mykonos, Greece	*Corallina* sp. and unidentified green algae	37.438991, 25.326656	MNCN 19487	−	−	PV475186	−
MYK 1.3					MNCN 19488	−	PV422755	PV475187	−
MYK 1.6					−	−	−	−	−
MYK 1.7					−	−	−	−	−
MYK 2					MNCN 19489	PV435825	PV422756	PV475190	PV422783
ELA 1.1	**Lineage 3**	Elafonisi Beach, Crete, Greece	*Corallina* sp. and unidentified green algae	35.271162, 23.541309	MNCN 19490	PV435828	−	PV475193	−
ELA 1.3					MNCN 19491	PV435826	PV422757	PV475191	−
ELA 1.6					−	−	−	−	−
ELA 1.7					−	−	−	−	−
ELA 1.8					−	−	−	−	−
CHA 1.6		Nea Chora Beach, Crete, Greece	*Corallina* sp. and unidentified green algae	35.51374, 24.00602	−	−	−	−	−
CHA 1.7					−	−	−	−	−
CHA 1.8					−	−	−	−	−
KED 1.1		Kedrodasos Beach, Crete, Greece	*Corallina* sp. and unidentified green algae	35.26858, 23.56310	MNCN 19492	−	PV422761	PV475188	PV422773
KED 1.2	**Lineage 4**	Kedrodasos Beach, Crete, Greece	*Corallina* sp. and unidentified green algae	35.26858, 23.56310	MNCN 19493	PV435832	PV422750	PV475181	PV422777
ELA 1.5		Elafonisi Beach, Crete, Greece	*Corallina* sp. and unidentified green algae	35.271162, 23.541309	MNCN 19494	PV435833	PV422751	PV475182	PV422778
ELA 2				MNCN 19495	PV435830	PV422759	PV475198	PV422782
MYK 1.5		Megali Ammos Beach, Mykonos, Greece	*Corallina* sp. and unidentified green algae	37.438991, 25.326656	MNCN 19496	PV435836	PV422762	PV475195	PV422770
CHA 1.2		Nea Chora Beach, Crete, Greece	*Corallina* sp. and unidentified green algae	35.51374, 24.00602	MNCN 19497	PV435829	PV422758	PV475199	−
CHA 1.3				MNCN 19498	PV435837	PV422763	PV475196	PV422771
CHA 1.1	**Not assigned**	Nea Chora Beach, Crete, Greece	*Corallina* sp. and unidentified green algae	35.51374, 24.00602	MNCN 19499	PV435835	PV422754	PV475197	PV422781
CHA 1.4					MNCN 19500	PV435831	PV422760	PV475183	PV422775
CHA 1.5					MNCN 19501	PV435827	−	PV475192	PV422774
ELA 1.2		Elafonisi Beach, Crete, Greece	*Corallina* sp. and unidentified green algae	35.271162, 23.541309	MNCN 19502	PV435834	PV422752	PV475180	PV422780

### ﻿Molecular analyses

Genomic DNA was extracted from a 2–3 segment section from 20 specimens, using the Speedtools DNA extraction kit (Biotools), following the manufacturer’s protocol. Molecular markers commonly used in other phylogenetic and species delimitation studies consisted of fragments of the nuclear *28S rRNA* (*28S*, ~531 bp) and *18S rRNA* (*18S*, ~1800 bp) and the mitochondrial markers *16S rRNA* (*16S*, ~470 bp) and *cytochrome c oxidase subunit I* (*COI*, ~650 bp), were amplified by polymerase chain reaction (PCR). Primers *28Sa* and *28Srd5b* (Edgecombe and Giribet 2006) were used to amplify the *28S rRNA* fragment. For *18S rRNA*, three overlapping pairs of primers were used: *18S1F*-*18S4R*, *18S3F*-*18Sbi*, and *18Sa2.0*- *18S9R* (Giribet et al. 1996; Whiting et al. 1997). Primers *16SarL* and *16SbrH* (Palumbi 1996) were used to amplify *16S rRNA*, and the modified primers with inosine *jgLCO1490* and *jgHCO2198* (Geller et al. 2013) were employed to amplify in all specimens. The PCR reactions consisted of 1.5 μl of DNA template in 19.5 μl reaction volume containing 12 μl of RED Taq polymerase master mix (VWR), 5 μl of H_2_O, and 0.5 μl of each of 10μM primers. In some cases, 0.8 μl of 10 mM bovine serum albumin (BSA) was added to stabilise enzymes during the process, bringing the final volume to 20.3 μl. The PCR protocols used were the same as those employed in previous studies on the family Syllidae (Álvarez-Campos et al. 2017b; Moreno-Martín et al. 2023; del Olmo et al. 2024).

Sanger sequencing was conducted by Macrogen Spain (Madrid, ES) (https://dna.macrogen.com/). All sequences were edited using GENEIOUS Prime 2025.0.2 (https://www.geneious.com/), to remove primers, and were deposited in the NCBI database (http://www.ncbi.nlm.nih.gov/genbank/).

### ﻿Phylogenetic analysis

The phylogenetic relationships among the collected specimens were studied by combining the newly obtained sequences with 143 additional sequences available in GenBank (https://www.ncbi.nlm.nih.gov/genbank/) (Suppl. material 1). A total of 29 species of the genus *Syllis* were also included to determine the phylogenetic position of the different Mediterranean population of *S. prolifera*, together with 16 species of some other genera from the subfamily Syllinae (*Trypanosyllis* Claparède, 1864; *Eurysyllis* Ehlers, 1864; *Ramisyllis* Glasby, Schroeder & Aguado, 2012; *Haplosyllis* Langerhans, 1879; *Branchiosyllis* Ehlers, 1887; and *Plakosyllis* Hartmann-Schröder, 1956), used as outgroups. The best evolutionary model for each amplified gene was selected using jModeltest2 (Darriba et al. 2012), following the Akaike Information Criterion (AIC) (Akaike 1974). The best evolutionary model for each gene was identified as the General Time Reversible model with gamma distribution and a proportion of invariable sites (GTR+G+I). The sequences of each gene were aligned using MAFFT (Katoh and Standley 2013) under default parameters and then concatenated using SeaView v. 5.1 (Gouy et al. 2021).

Maximum Likelihood (ML) phylogenetic analysis of the concatenated sequence dataset was performed using raxmlGUI 2.0.13 (Edler et al. 2021), employing the GTR+G+I model with 500 bootstrap replicates to estimate support values. Bayesian Inference (BI) analysis was performed using MrBayes v. 3.2.7 (Ronquist et al. 2012), with four Markov chains starting from a random tree, run simultaneously for 50 million generations, sampling one tree every 5,000 generations (samplefreq = 5000). Additionally, the first 25% of the trees were discarded as burn-in (burninfrac = 0.25) after evaluating convergence using Tracer v. 1.7.2 (Rambaut et al. 2018). The resulting phylogenetic trees from both analyses were exported and visualised using FigTree v. 1.4.4 (http://tree.bio.ed.ac.uk/software/figtree/). Final editing of the phylogenetic trees, species delimitation trees and haplotype networks were performed using Inkscape v. 1.4 (https://inkscape.app/es/).

### ﻿Species delimitation and haplotype networks

*COI* and *16S* dataset were analysed separately using three different methods for species delimitation analyses: Automatic Barcode Gap Discovery (ABGD), General Mixed-Yule Coalescent (GMYC), and Bayesian Poisson Tree Process (bPTP), the latter in two variations (bPTP_BI and bPTP_ML). All species delimitation models were applied to simplified data set matrices including only one representative for each haplotype, as recommended by a previous study (Magoga et al. 2021). The ABGD method was run on its web server (https://bioinfo.mnhn.fr/abi/public/abgd/abgdweb.html) using the Kimura 2-parameter model (K2P) (Kimura 1980) and default parameters. For GMYC and bPTP models, ultrametric trees were first generated using BEAST v. 1.10.4 (Suchard et al. 2018) via BEAUti v1.10.4 using the GTR+G+I model, an uncorrelated lognormal relaxed clock, and a coalescent constant size prior. MCMC chains ran for 50 million generations (sampling every 1,000; 25% burn-in), with convergence (ESS > 200) checked in Tracer v. 1.7.2. Resulting trees were summarised to a maximum clade credibility topology in TreeAnnotator v. 1.10.4 (Suchard et al. 2018) (25% burn-in, mean node heights) and subjected to single-threshold GMYC analysis using the web server interface (https://species.h-its.org/gmyc/). Additionally, bPTP analyses was performed using the web server (https://species.h-its.org/ptp/) with the best ML tree and 200,000 generations (25% burn-in).

Genetic distances within and between lineages of *S. prolifera* sequences identified in the phylogenetic analyses were estimated using the software MEGA 11.0.13 (Tamura et al. 2021). Distances were calculated using the Kimura 2-parameter (K2P) model (Kimura 1980) for the *COI* and *16S* markers separately. Although this model has not been reported as an appropriate model in DNA-barcoding studies (Srivathsan and Meier 2012), and it has not been confirmed as the model that best fits our data, we estimated the distances using it to allow further comparisons with other studies. Haplotype networks – used to illustrate evolutionary relationships between populations – were built using PopART v. 1.7 (Population Analysis with Reticulate Trees) (Leigh and Bryant 2015), applying the TCS algorithm (Clement et al. 2002).

## ﻿Results

### ﻿Phylogenetic reconstruction

**Figure 2.**
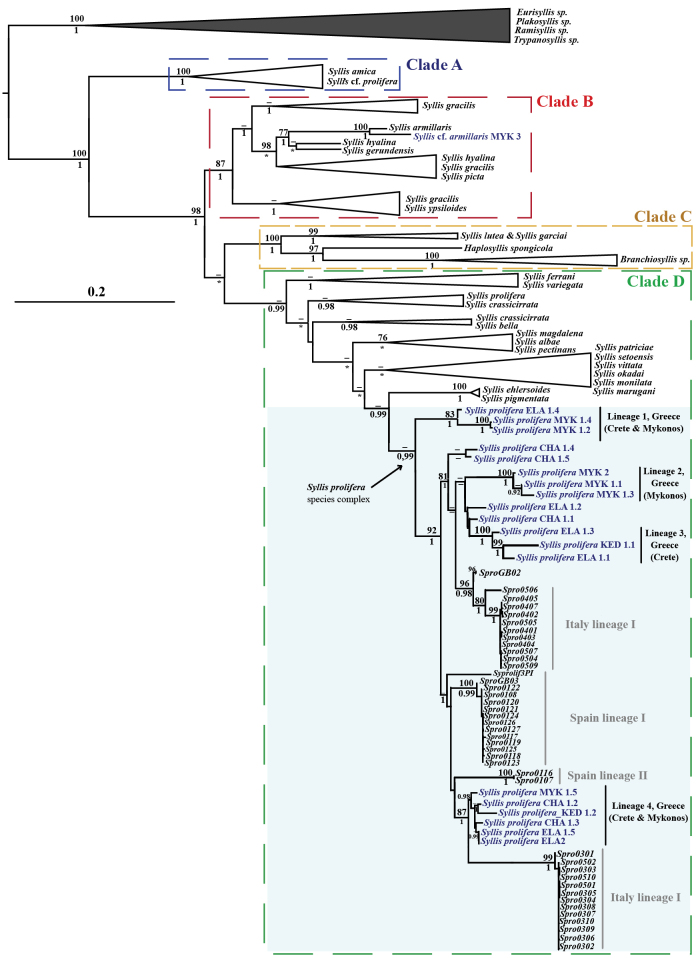
Phylogenetic relationships of *Syllis* and related genera based on concatenated molecular data. Tree obtained by the Maximum Likelihood method using the GTR+G+I model for the concatenated dataset (*COI*, *16S*, *28S*, *18S*), showing the phylogenetic relationships of the studied specimens and other closely related species. Numbers above the nodes indicate bootstrap support values equal to or greater than 75% (BS ≥ 75%), and numbers below indicate posterior probability values equal to or greater than 0.95 (PP ≥ 0.95). Dashes indicate lack of support in one of the analyses, and asterisks indicate that those clades were not recovered by the BI analysis (see Suppl. material 3). The names of the newly sequenced individuals are highlighted in blue. Within the *S. prolifera* complex, the newly defined lineages are shown in black, while the lineages defined in previous studies are outlined in grey.

Final alignments of all concatenated markers had a total length of 3,348 base pairs (bp). From the 163 specimens included in the analyses, 140 of them had sequences for the *COI* marker (624 bp), 110 for *16S* (421 bp), 98 for *18S* (1,990 bp), and 47 for *28S* (313 bp). ML and BI analyses yielded largely congruent topologies for the four-gene concatenated dataset, with minor differences observed in clade support values and some interspecific relationships (Fig. 2, Suppl. materials 2, 3). Both ML and BI analyses revealed that *Syllis* does not form a monophyletic group, with species from the genera *Haplosyllis* and *Branchiosyllis* clustering within the *Syllis* clade (Fig. 2, Suppl. materials 2, 3). Our analyses showed topologies with four major clades: Clade A (BS = 100%, PP = 1), which includes *S. amica* Quatrefages, 1866 and several specimens identified as Syllis cf. prolifera from brackish waters in Sardinia, Italy (del Olmo et al. 2024); Clade B (BS = 87%, PP = 1), which includes *Syllis* species reported within the *S. gracilis* species complex and related species (Álvarez-Campos et al. 2017a); Clade C (BS = 100%, PP = 1), which contains *S. lutea* (Hartmann-Schröder, 1960) and *S. garciai* (Campoy, 1982) together with *Haplosyllis* and *Branchiosyllis* species; and Clade D, only supported in the BI analysis (PP = 0.99) comprises the rest of *Syllis* species (Fig. 2, Suppl. materials 2, 3). Within clade D, there is a well-supported subclade in BI results (PP = 0.99) that contains the *S. prolifera* species complex (shaded on the tree). This complex contains four well-supported lineages with specimens from Mykonos and Crete together with other four individuals not included in any supported lineage (CHA 1.1, 1.4, 1.5, and ELA 1.2), as well as the five lineages previously identified by del Olmo et al. (2024). Our Greek lineage 1 includes only three individuals from Crete and Mykonos (BS = 83%, PP = 1) and is the sister group to the remaining lineages (BS = 92%, PP = 1). Lineage 2 only includes three individuals from Mykonos (BS = 100%, PP = 1) and appears closely related to lineage 3, which groups three individuals from Crete (BS = 100%, PP = 1). Finally, lineage 4 (PP = 0.98) clearly differentiated from the remaining lineages from Greece, includes six mixed individuals from all studied sites and is the sister group of a lineage from Italy.

### ﻿Species delimitation within the *S. prolifera* complex

**Figure 3.**
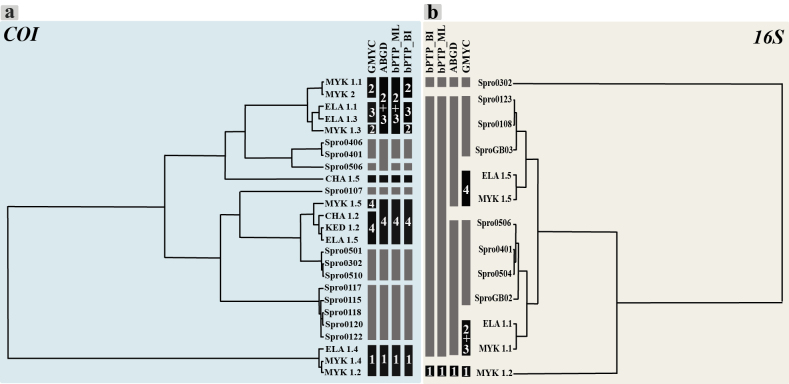
Ultrametric trees of the different models used for species delimitation in the *Syllis prolifera* complex using *COI* (**a**) and *16S* (**b**) haplotype datasets. Black bars indicate haplotype clusters/potential species supported by each analysis (see Suppl. materials 4–9) while grey bars indicate those from a previous study (del Olmo et al. 2024). Numbers within each bar correspond to the lineages (1–4) resulting from the phylogenetic analysis.

Species delimitation analyses of the *COI* marker using only the 25 haplotype sequences identified six or seven lineages and – 5 singletons (Fig. 3a). GMYC model identified seven lineages – four of them including specimens from Greece– and five singletons, one from Crete (CHA 1.5) and two from Mykonos (Fig. 3a, Suppl. material 4). Greek lineages 1 and 3 comprised the same specimens recovered by the phylogenetic analyses, lineage 2 included two specimens from Mykonos and lineage 4 contained three specimens from the different localities in Crete (Fig. 3a). ABGD model recovered six lineages – three from Greece – and two singletons, one of them being the Cretan CHA 1.5 (Fig. 3a, Suppl. material 5). Greek lineages 1 and 4 included the same individuals than the phylogenetic analyses, and lineages 2 and 3 grouped together as a single lineage (Fig. 3a). Similarly, the ML variant of the bPTP model delimited the same six lineages as the previous model and three singletons, including again CHA 1.5 (Fig. 3a, Suppl. material 6). Once more, Greek lineages 1 and 4 included the same individuals than the phylogenetic analyses, and lineages 2 and 3 grouped together as a single lineage. Finally, the BI variant of the bPTP analysis identified seven lineages –four including specimens from Greece – and four singletons (Fig. 3a, Suppl. materials 6). In this case, Greek lineages 1, 3, and 4 comprised the same individuals as in the phylogenetic analyses. The lineages from Italy and Spain previously identified by del Olmo et al. (2024) were the same in all the delimitation analyses of *COI*, except for specimen Spro0506 (Italian lineage I), that was considered a singleton in GMYC and both bPTP analyses (Fig. 3a, Suppl. materials 4, 6).

On the other hand, the species delimitation analyses for the *16S* marker using only the 13 haplotype sequences identified one to four lineages and two singletons (Fig. 3b). The species delimitation results for the *16S* haplotypes were exactly the same for the GMYC and ABDG models, both considering a unique lineage except for individuals Spro0302 and MYK 1.2, considered as singletons (Fig. 3b, Suppl. materials 7, 8). Likewise, ML and BI variants of bPTP identified the same two singletons and four lineages, two of them containing specimens from Greece (Fig. 3b, Suppl. materials 9). Greek Lineage 4 comprised the same specimens recovered by the phylogenetic analyses, while lineages 2 and 3 appeared together as a single lineage (Fig. 3b).

### ﻿Genetic distances and haplotype networks

Pairwise genetic distances (K2P) for the *S. prolifera* species complex were calculated between the nine supported lineages identified by phylogenetic analysis. The K2P distance for *COI* ranged from 3.2% ± 0.6 to 25.7% ± 2.6, with the lowest distance between the lineage 2 (Mykonos) and lineage 3 (Crete), and the largest one between lineage 1 (Crete and Mykonos) and lineage I from Italy (Table 2). The genetic distances between the Cretan specimen CHA 1.5 and the rest of lineages, ranged from 15.4% ± 2.0 to 24.7% ± 2.7. Intralineage distances for the *COI* ranged from 0 to 2.8% ± 0.5, with the lowest in the Spanish lineage II and the highest within the Greek lineage 2 (Table 2).

**Table 2.** Genetic distances among lineages of the *Syllis prolifera* complex. Intraspecific (in bold, *COI*: above; *16S*: below) and interspecific genetic distances for the *COI* (lower left) and *16S* (upper right) markers among the different lineages delimited within the *S. prolifera* complex, calculated using the Kimura 2-parameter (K2P) model. Distances are expressed as percentages. Dashes indicate the absence of sequences for that marker.

	Lineage 1 Greece	Lineage 2 Greece	Lineage 3 Greece	Lineage 4 Greece	CHA 1.5	ELA 1.2	Italy lineage I	Italy lineage II	Spain lineage I	Spain lineage II
Lineage 1, Greece (Crete & Mykonos)	**0.7 ± 0.3 / 0.3 ± 0.2**	24.5 ± 2.8	23.2 ± 2.8	24.7 ± 2.9	–	25.5 ± 3.0	25.1 ± 2.8	45.6 ± 6.0	25.9 ± 3.0	–
Lineage 2, Greece (Mykonos)	21.0 ± 2.1	**2.8 ± 0.6 / 1.2 ± 0.5**	0.8 ± 0.5	7.4 ± 1.4	–	2.6 ± 0.8	4.9 ± 1.1	44.3 ± 5.9	7.8 ± 1.5	–
Lineage 3, Greece (Crete)	20.5 ± 2.1	3.2 ± 0.6	**0.9 ± 0.4 / 0.0 ± 0.0**	7.5 ± 1.5	–	1.2 ± 0.5	4.4 ± 1.1	43.7 ± 5.9	7.2 ± 1.5	–
Lineage 4, Greece (Crete & Mykonos)	22.8 ± 2.2	18.0 ± 2.0	18.4 ± 2.0	**1.2 ± 0.3 / 0.6 ± 0.3**	–	8.9 ± 1.6	8.9 ± 1.6	44.6 ± 6.1	4.5 ± 1.1	–
CHA 1.5 (Crete)	24.7 ± 2.7	15.4 ± 2.0	15.4 ± 2.1	17.4 ± 2.2	–/–	–	–	–	–	–
ELA 1.2 (Crete)	–	–	–	–	–	–/–	6.4 ± 1.3	43.7 ± 5.9	9.1 ± 1.7	–
Italy lineage I	25.7 ± 2.5	13.5 ± 1.6	13.4 ± 1.7	20.1 ± 2.1	15.8 ± 2.0	–	**1.4 ± 0.2 / 0.4 ± 0.2**	42.0 ± 5.6	8.0 ± 1.5	–
Italy lineage II	23.4 ± 2.3	20.0 ± 2.3	20.3 ± 2.3	4.8 ± 0.9	17.6 ± 2.3	–	21.1 ± 2.2	**0.2 ± 0.1** / –	42.4 ± 5.7	–
Spain lineage I	24.2 ± 2.4	16.9 ± 2.1	17.3 ± 1.9	15.2 ± 1.7	16.2 ± 2.0	–	19.0 ± 2.0	15.2 ± 1.7	**0.2 ± 0.1 / 0.3 ± 0.2**	–
Spain lineage II	24.3 ± 2.3	19.3 ± 1.9	17.7 ± 2.0	15.0 ± 1.7	18.2 ± 2.2	–	19.5 ± 2.0	14.4 ± 1.7	17.9 ± 1.6	**0.0 ± 0.0** /–

On the other hand, the K2P distances for *16S* ranged from 0.8% ± 0.5 to 45.6% ± 6.2, with the lowest again between the Greek lineages 2 and 3, and the largest between the Greek lineage 1 and the Italian lineage II (Table 2). The variation of genetic distances between the Cretan individual ELA 1.2 and the remaining lineages ranged from 6.4% ± 1.3 to 43.7 ± 5.9 (Table 2). The intralineage distances ranged from 0 to 1.2% ± 0.5, with the lowest being within the Greek lineage 3 and the Spanish lineage II, and the highest distance again being within the Greek lineage 2 (Table 2).

**Figure 4.**
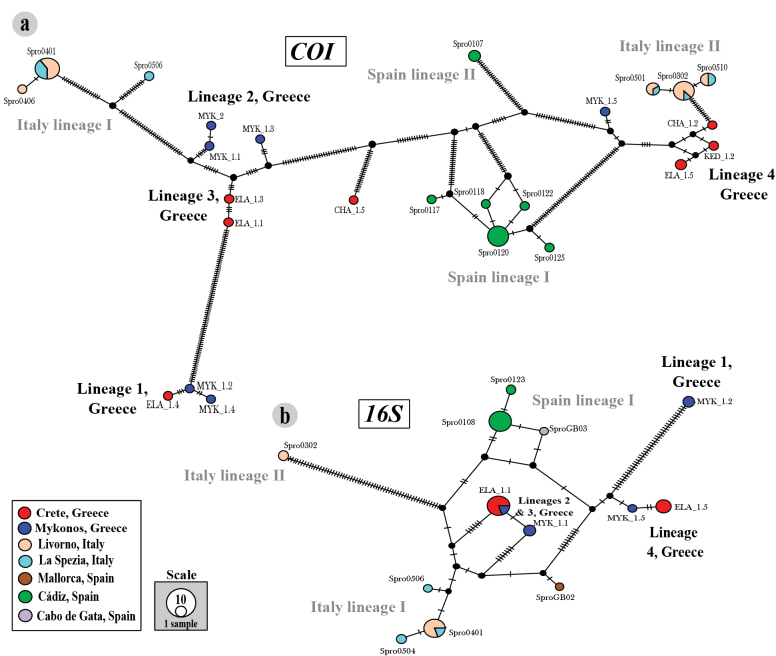
Haplotype networks for *COI* (**a**) and *16S* (**b**) markers, and distribution areas of the different lineages within the *S. prolifera* complex. Each locality is represented by a different colour (see legend), and the size of the circles, which represent distinct haplotypes, indicates the number of individuals included (see scale). Newly defined lineages in previous sections are shown in black, while those defined in a previous study (del Olmo et al. 2024) are shown in grey.

To explore the genetic differences among sampled *S. prolifera* populations in the eastern Mediterranean and the previously studied ones from the western Mediterranean and Gulf of Cádiz, we also inferred the TCS haplotype networks including 54 sequences for *COI* and 35 for *16S*. Both the *COI* and *16S* haplotype networks recovered multiple differentiated groups, some of them corresponding to the phylogenetic lineages, suggesting that they are indeed different putative species (Fig. 4).

The haplotype network analysis of *COI* marker identified eight highly differentiated groups – four including specimens from Greece and four from the west Mediterranean – and four singletons separated by several mutational steps – MYK 1.3, MYK 1.5, and CHA 1.5 from Greece and the Italian Spro0506 (Fig. 4a). The Greek lineage 2, excluding specimen 1.3, was separated from lineage 3 by 16 mutation steps. As in most of the analyses of previous sections, the specimens MYK 1.3 and MYK 1.5 were clearly distinct from their closest lineages by 13 and 8 mutational steps, respectively (Fig. 4a). Similarly, the Cretan specimen CHA 1.5 was also separated from the rest of lineages by more than 40 mutational steps (Fig. 4a). All the groups that included Italian and Spanish specimens were the same identified in a previous study (del Olmo et al. 2024). A total of 160 polymorphic sites (25.6%) were found in this data set.

The haplotype network analysis of *16S* marker identified six well-differentiated groups – three of them including individuals from Greece and three from the western Mediterranean – and one singleton (Fig. 4b). The Greek lineage 1 is only represented by one haplotype, differentiated from the rest by more than 40 mutations and lineage 4 includes two different haplotypes separated by two mutational steps (Fig. 4b). Similarly, the third group includes two different haplotypes separated by a single mutational step, with specimens belonging to the phylogenetic lineages 2 and 3 (Fig. 4b). Again, all the groups that included Italian and Spanish specimens were the same as those identified in a previous study (del Olmo et al. 2024). A total of 86 polymorphic sites (20.4%) were found in this data set.

Overall, the haplotype networks do not reveal a clear division between eastern and western lineages, as some western lineages showed larger mutational divergence from each other than to the eastern lineages (e.g. Italian lineage I) (Fig. 4a). However, the Greek lineages exhibit geographical structure, as individuals from Mykonos were separated from those collected in Crete, except for one specimen in Lineage 1 that could be actually considered as a sampling artefact (Figs 1, 4a).

### ﻿Morphological study

**Figure 5.**
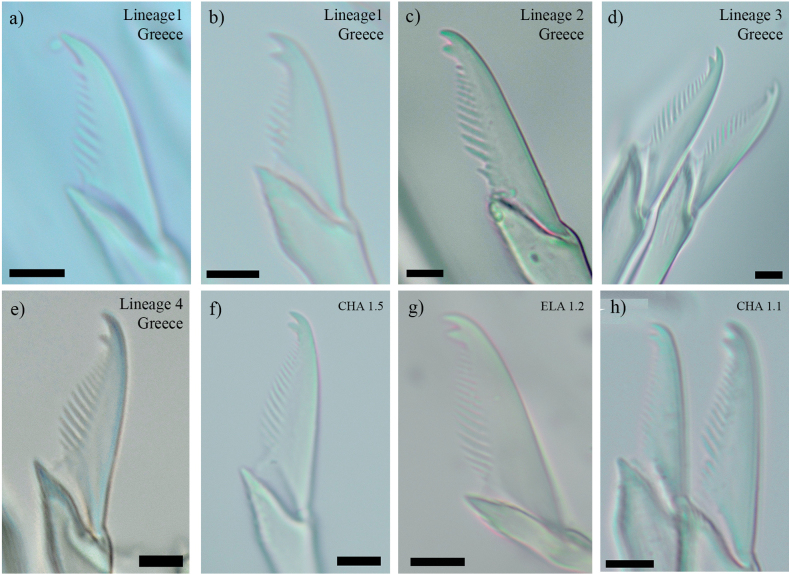
Bright-field optical microscopy photographs of the posterior compound chaetae of studied specimens in Greece. **a.** MYK 1.4 (Lineage 1, Greece); **b.** ELA 1.4 (Lineage 1, Greece); **c.** MYK 2 (Lineage 2, Greece); **d.** ELA 1.1 (Lineage 3, Greece); **e.** KED 1.2 (Lineage 4, Greece); **f.** CHA 1.5 (no lineage); **g.** ELA 1.2 (no lineage); **h.** CHA 1.1 (no lineage). Scale bars: 5 μm.

**Figure 6.**
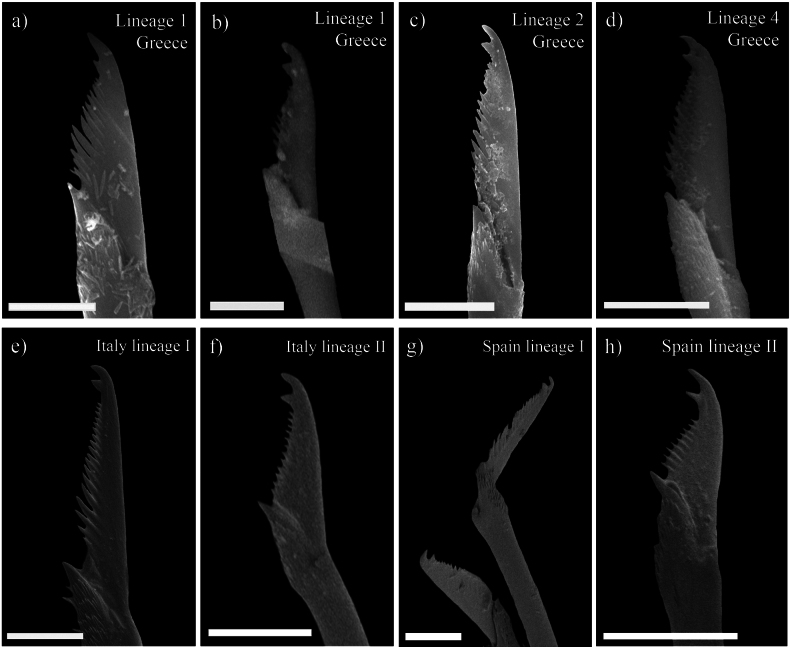
Scanning electron micrographs of the posterior compound chaetae of the lineages delimited within the *Syllis prolifera* complex, including main lineages from Greece and unpublished images of western Mediterranean lineages (del Olmo et al. 2024). **a.** MYK 1.4 (Lineage 1, Greece); **b.** ELA 1.4 (Lineage 1, Greece); **c.** MYK 1.1 (Lineage 2, Greece); **d.** MYK 1.5 (Lineage 4, Greece); **e.** Spro0507 (Italy lineage I); **f.** Spro0307 (Italy lineage II); **g.** Spro0118 (Spain lineage I); **h.** Spro0116 (Spain lineage II). Scale bars: 10 μM.

In agreement with our molecular studies (see above), the morphological examination revealed some few differences between some of the lineages of *S. prolifera* analysed in Greece. Specifically, some small differences were observed in the length of proximal and distal teeth of most posterior compound chaetae. Thus, individuals from lineages 1 and 4 presented a distal tooth slightly longer than the proximal one, whereas specimens from lineages 2 and 3, as well as the specimens from Crete not included in any lineage the molecular analyses had both teeth similar in length (Figs 5a–h, 6a–d). Nevertheless, within lineage 1, one specimen was found presenting teeth of the same length (Figs 5b, 6b). On the other hand, the morphological comparisons with specimens from lineages defined in the previous study of *S. prolifera* (del Olmo et al. 2024) also showed some similarities with some of the new Greek lineages. Thus, lineage I from Italy is characterised by posterior blades with proximal and distal teeth of same length and long spines on margin, as in the case of Greek lineages 2 and 3 (Fig. 6e). The remaining lineages from Spain and Italy also presented blades with teeth of similar length but the spines on margin are very short, a feature that can easily differentiate them from the Greek lineages (Fig. 6f–h).

## ﻿Discussion

Our integrative analysis using molecular and morphological data from several eastern Mediterranean populations of *Syllis prolifera* provides robust evidence that this taxon constitutes a complex of different species, in agreement with the previous study on the western Mediterranean populations (del Olmo et al. 2024). The phylogenetic and species delimitation analyses have shown that there are at least three clearly distinct and well-supported lineages in Greece (Figs 2, 3, Suppl. materials 2–6), that would probably increase with a more exhaustive sampling regime in the Aegean Sea.

These results also agreed with the genetic distances observed within and between some of the lineages identified. For *COI*, intralineage divergence showed very low rates, always below 1.5%, except for the lineage 2 from Mykonos, where differences of 2.8% ± 0.6 were observed (Table 2). These intralineage values consistently remained much lower than K2P interlineage ones, which ranged from 13.4% ± 1.7 to 25.7% ± 2.6, except in the comparison of lineages 2 and 3, with a distance of 3.2% ± 0.6, and between lineage 4 and lineage II from Italy, with a distance of 4.8% ± 0.9 (Table 2). For all except for those lineages, our results fall in the range of those of the previous study in *S. prolifera*, with 10.5 to 27.4% (del Olmo et al. 2024), and also in other closed related species as *S. gracilis*, with 17.5 to 36.6% (Álvarez-Campos et al. 2017a). In addition, similar ranges have been proposed in other Syllidae genera and also in other polychaete families (Nygren and Pleijel 2011; Neal et al. 2014; Álvarez-Campos et al. 2017b; Aguado et al. 2019; Grosse et al. 2020; Hektoen et al. 2022; Teixeira et al. 2025). Actually, the limit for *COI* genetic distance values considered as sufficiently significant to classify two groups as different species has been established in polychaetes in the 10% (Kvist 2016). On the other hand, the interlineage K2P distances calculated for our *16S* dataset are also higher than the intralineage ones, and similar to those described *S. prolifera* (7.7–38.6%, del Olmo et al. 2024) and in other polychaetes (Bastrop et al. 1998; Miglietta et al. 2010). However, once again the differences between lineages 2 and 3 are in the same range than the intralineages ones, with a variation in *16S* of only 0.8% ± 0.5 (Table 2). Haplotype network of *16S* also showed a blurred boundary between these two lineages, differing only by one mutation (Fig. 4). All these results together with the fact that several of the species delimitation analyses considered both as a single lineage, did not allow us to unequivocally propose lineages 2 and 3 as different taxonomic entities.

Furthermore, the detailed morphological examination of the Greek specimens, also allowed us to recognise at least two lineages within *S. prolifera* complex, by describing subtle details on their posterior chaetae (Figs 5, 6). However, some of the lineages turned out to be impossible to distinguish with any of the considered morphological features, i.e. within lineages 1 and 4 and within lineages 2 and 3, as well as between them and some of the Italian and Spanish lineages (Fig. 6). In addition, the molecular singleton specimen CHA 1.5 has the posterior chaetae quite similar to those observed in lineages 1 and 4 (Fig. 5a, b, e, f). While previous studies highlighted differences in the pattern of dorsal cirri between different lineages of *S. prolifera* (del Olmo et al. 2024), such differences were not identified between different lineages sampled in the Aegean Sea. Altogether, our results suggest that *S. prolifera* complex comprises between seven and nine lineages in the Mediterranean Sea (two and four in Greece and 5 in Spain and Italy), both cryptic and pseudocryptic, further complicating the delineation of species boundaries. Moreover, the number of species within *S. prolifera* complex could increase substantially with the expansion of sampling efforts in this and other regions of the Mediterranean Sea. Interestingly, the ecological adaptation indicated in the previous study of *S. prolifera* or in other Syllidae complexes (del Olmo et al. 2024; Langeneck et al. 2020; Álvarez-Campos et al. 2017a, b), did not provide clear boundaries among our Greek lineages, since all specimens were found in coastal shallow habitats with similar algae substrates (Table 1, Suppl. material 1). Besides, part of our results reveal evidence of phylogeographic structuring since none of the Greek lineages show shared haplotypes between Mykonos and Crete islands (Fig. 4a). Moreover, the clear separation showed between the Cretan lineage 4 and the Italian lineage II suggests that the divergence between these two lineages might have been driven by phylogeographic barriers between the western and the western Mediterranean Sea, as it was also recently proposed for the species included in the Mediterranean nereid complexes *Perinereis cultrifera* and *P. rullieri* (Teixeira et al. 2025).

In general, our results suggest that eastern Mediterranean coasts probably host a higher percentage of endemic syllid species with reproductively isolated populations from the western part, instead of species with wide distributions, as it has been shown not only in the case of *S. prolifera* but also in other worldwide distributed *Syllis* species (Álvarez-Campos et al. 2017a). In fact, all the reported Syllidae species from the Aegean and Levant Seas are thought to be widely distributed species, not only in the Mediterranean Sea, but also in other oceans as in the Indo-Pacific (Çinar and Ergen 2002; Çinar et al. 2003, 2014, 2024; San Martín 2003; Rousou et al. 2023; Toso et al. 2024). In the case of *S. prolifera*, material from the type locality in Nice (France), together with some other intermediate localities in the Mediterranean Sea would be essential in order to clarify the populations dynamics of the different reported lineages. In addition, formal taxonomic descriptions are also essential when working with cryptic and pseudocryptic species complexes for their recognition and inclusion in the already diverse species inventories of Mediterranean polychaetes (Rousou et al. 2023; Toso et al. 2024). Nevertheless, the present study represents a significant step towards resolving the speciation patterns of *S. prolifera* complex and highlights the importance of integrative approaches for uncovering hidden biodiversity in marine invertebrates, which can also contribute to their proper conservation.

## ﻿Conclusions

Our findings emphasise that relying exclusively on morphological traits leads to a significant underestimation of marine biodiversity, particularly within taxa with difficult taxonomy or problems of misinterpretations of these features, as syllid polychaetes. The species *Syllis prolifera*, previously considered a single, widely distributed taxon across the Mediterranean Sea, were shown to comprise five pseudocryptic species in the western Mediterranean and our study now adds evidence for the presence of at least three additional ones from the eastern basin. These newly identified lineages exhibit geographically restricted distributions in the two Greek islands and are clearly distinct from their western counterparts. For some of the lineages, we identified subtle but consistent morphological differences – such as variations in the length of teeth and spines of posterior chaetae – that align with the molecular data. Nonetheless, some of the proposed Greek lineages remain difficult to delimit morphologically pointing to the presence of cryptic speciation within this complex. Overall, our results highlight the necessity of adopting integrative methods for species delimitation, both to resolve taxonomic uncertainties and to accurately assess the marine diversity, especially difficult in environments with complex biogeographic patterns as in the eastern Mediterranean Sea.

## ﻿Additional information

### Use of AI

No use of AI was reported.

### Author ORCIDs

M. Rosario Martín-Hervás https://orcid.org/0000-0002-9673-2673

Irene del Olmo https://orcid.org/0000-0001-5448-1039

Patricia Álvarez-Campos https://orcid.org/0000-0001-9472-2378

### Data availability

All of the data that support the findings of this study are available in the main text or Supplementary Information.

## Supplementary materials

Supplementary material 1 Sequences used in the phylogenetic analysis Data type xlsx Explanation note List of specimens with species names, collection localities, substrates, geographic coordinates, catalogue numbers, and GenBank accession numbers. Dashes (–) indicate absence of data. Asterisks (*) indicate unpublished sequences. https://binary.pensoft.net/file/1493555This dataset is made available under the Open Database License (http://opendatacommons.org/licenses/odbl/1.0/). The Open Database License (ODbL) is a license agreement intended to allow users to freely share, modify, and use this Dataset while maintaining this same freedom for others, provided that the original source and author(s) are credited.Fernando Belvis, M. Rosario Martín-Hervás, Irene del Olmo, Víctor Ruiz, Patricia Álvarez-Campos

Supplementary material 2 Phylogenetic tree of the *S. prolifera* complex (ML, GTR+G+I) Data type svg Explanation note Most likely phylogenetic tree obtained by the ML method with the GTR+G+I model for all sequenced markers (*COI*, *16S*, *28S*, *18S*). Posterior probabilities are shown next to each node. The clade containing the specimens used as the outgroup is highlighted in black. In the *S. prolifera* complex, the new lineages are shown in black, while the lineages defined in previous studies are outlined in grey. https://binary.pensoft.net/file/1493556This dataset is made available under the Open Database License (http://opendatacommons.org/licenses/odbl/1.0/). The Open Database License (ODbL) is a license agreement intended to allow users to freely share, modify, and use this Dataset while maintaining this same freedom for others, provided that the original source and author(s) are credited.Fernando Belvis, M. Rosario Martín-Hervás, Irene del Olmo, Víctor Ruiz, Patricia Álvarez-Campos

Supplementary material 3 Phylogenetic tree of the *S. prolifera* complex (BI, GTR+G+I) Data type svg Explanation note Most likely phylogenetic tree obtained by the BI method with the GTR+G+I model for all sequenced markers (*COI*, *16S*, *28S*, *18S*). Posterior probabilities are shown next to each node. The clade containing the specimens used as the outgroup is highlighted in black. In the *S. prolifera* complex, the new lineages are shown in black, while the lineages defined in previous studies are outlined in grey. https://binary.pensoft.net/file/1493557This dataset is made available under the Open Database License (http://opendatacommons.org/licenses/odbl/1.0/). The Open Database License (ODbL) is a license agreement intended to allow users to freely share, modify, and use this Dataset while maintaining this same freedom for others, provided that the original source and author(s) are credited.Fernando Belvis, M. Rosario Martín-Hervás, Irene del Olmo, Víctor Ruiz, Patricia Álvarez-Campos

Supplementary material 4 Ultrametric tree resulting from the species delimitation analysis under the GMYC model for *COI* haplotypes of *S. prolifera* individuals Data type svg Explanation note Posterior probabilities are shown next to each node. https://binary.pensoft.net/file/1493558This dataset is made available under the Open Database License (http://opendatacommons.org/licenses/odbl/1.0/). The Open Database License (ODbL) is a license agreement intended to allow users to freely share, modify, and use this Dataset while maintaining this same freedom for others, provided that the original source and author(s) are credited.Fernando Belvis, M. Rosario Martín-Hervás, Irene del Olmo, Víctor Ruiz, Patricia Álvarez-Campos

Supplementary material 5 Species delimitation results for ABGD model for the *COI* haplotypes from *S. prolifera* individuals Data type tif https://binary.pensoft.net/file/1493559This dataset is made available under the Open Database License (http://opendatacommons.org/licenses/odbl/1.0/). The Open Database License (ODbL) is a license agreement intended to allow users to freely share, modify, and use this Dataset while maintaining this same freedom for others, provided that the original source and author(s) are credited.Fernando Belvis, M. Rosario Martín-Hervás, Irene del Olmo, Víctor Ruiz, Patricia Álvarez-Campos

Supplementary material 6 Trees resulting from species delimitation under the bPTP model for *COI* haplotypes of *S. prolifera* specimens Data type tif Explanation note The tree on the left corresponds to the bPTP_ML analysis, while the tree on the right corresponds to the bPTP_BI analysis. https://binary.pensoft.net/file/1493560This dataset is made available under the Open Database License (http://opendatacommons.org/licenses/odbl/1.0/). The Open Database License (ODbL) is a license agreement intended to allow users to freely share, modify, and use this Dataset while maintaining this same freedom for others, provided that the original source and author(s) are credited.Fernando Belvis, M. Rosario Martín-Hervás, Irene del Olmo, Víctor Ruiz, Patricia Álvarez-Campos

Supplementary material 7 Ultrametric tree resulting from the species delimitation analysis under the GMYC model for *16S* haplotypes of *S. prolifera* individuals Data type svg Explanation note Posterior probabilities are shown next to each node. https://binary.pensoft.net/file/1493561This dataset is made available under the Open Database License (http://opendatacommons.org/licenses/odbl/1.0/). The Open Database License (ODbL) is a license agreement intended to allow users to freely share, modify, and use this Dataset while maintaining this same freedom for others, provided that the original source and author(s) are credited.Fernando Belvis, M. Rosario Martín-Hervás, Irene del Olmo, Víctor Ruiz, Patricia Álvarez-Campos

Supplementary material 8 Species delimitation results for ABGD model for the *16S* haplotypes from *S. prolifera* individuals Data type tif https://binary.pensoft.net/file/1493562This dataset is made available under the Open Database License (http://opendatacommons.org/licenses/odbl/1.0/). The Open Database License (ODbL) is a license agreement intended to allow users to freely share, modify, and use this Dataset while maintaining this same freedom for others, provided that the original source and author(s) are credited.Fernando Belvis, M. Rosario Martín-Hervás, Irene del Olmo, Víctor Ruiz, Patricia Álvarez-Campos

Supplementary material 9 Trees resulting from species delimitation under the bPTP model for *16S* haplotypes of *S. prolifera* specimens Data type tif Explanation note The tree on the top corresponds to the bPTP_ML analysis, while the tree on the bottom corresponds to the bPTP_BI analysis. https://binary.pensoft.net/file/1493563This dataset is made available under the Open Database License (http://opendatacommons.org/licenses/odbl/1.0/). The Open Database License (ODbL) is a license agreement intended to allow users to freely share, modify, and use this Dataset while maintaining this same freedom for others, provided that the original source and author(s) are credited.Fernando Belvis, M. Rosario Martín-Hervás, Irene del Olmo, Víctor Ruiz, Patricia Álvarez-Campos
