# Supplementary figures and images for: ﻿New insights into the Syllis prolifera species complex from the eastern Mediterranean Sea
Source: Zookeys. 2025 Dec 16;1264:159–81. doi: 10.3897/zookeys.1264.170411 (PMC12728531; doi:10.3897/zookeys.1264.170411)

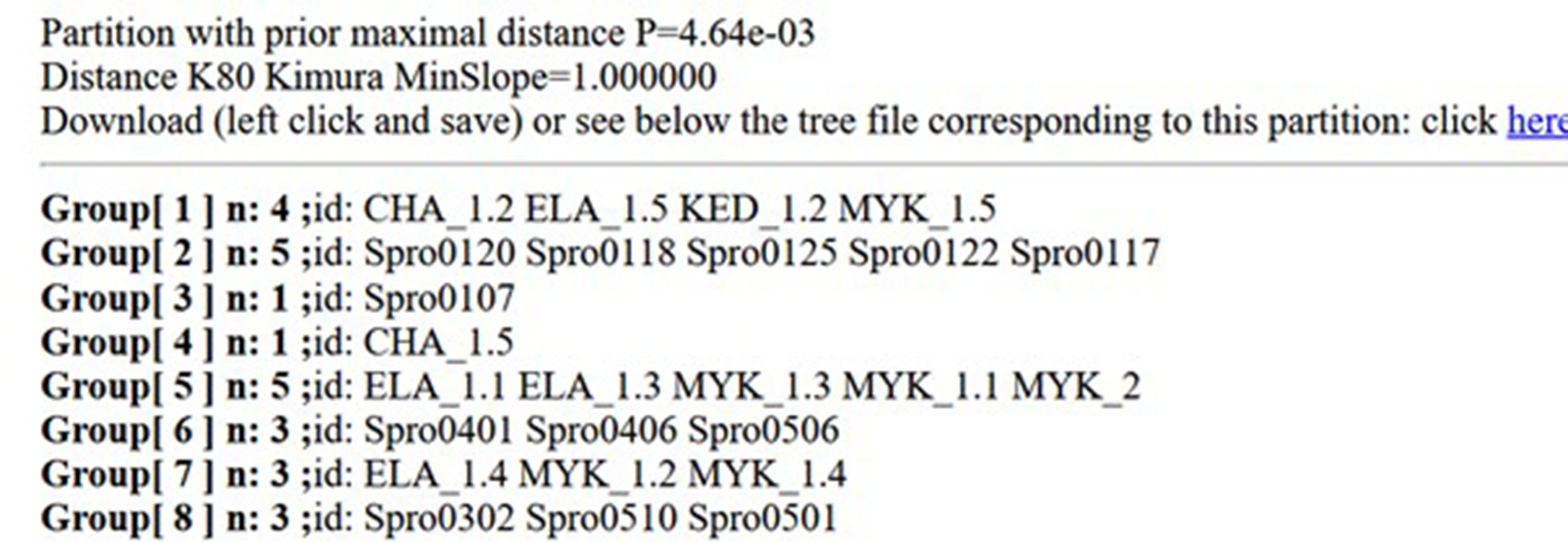

Supplement: Supplementary material 5 — Species delimitation results for ABGD model for the COI haplotypes from S. prolifera individuals [file zookeys-1264-159_article-170411__-s005.tif]

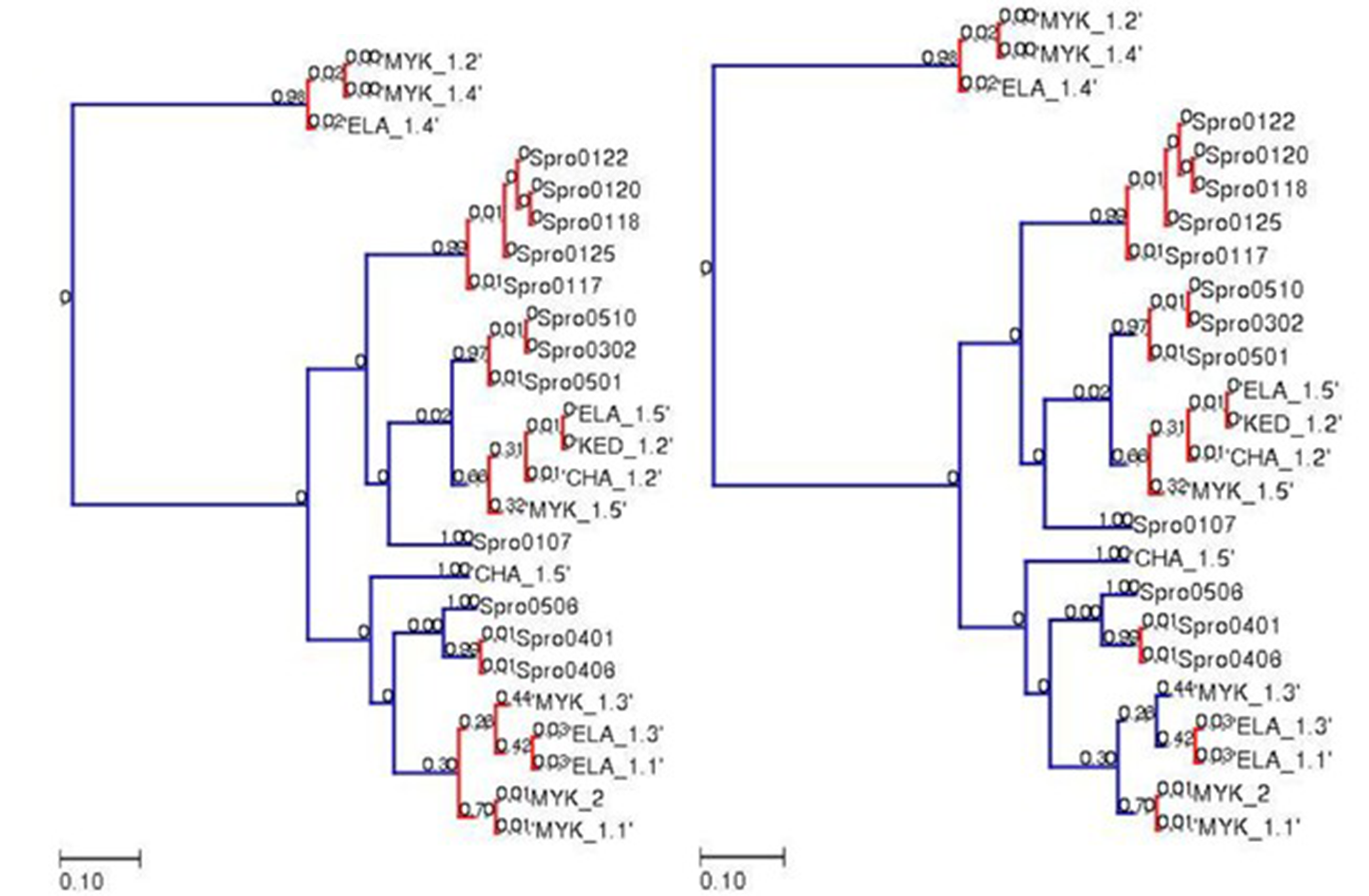

Supplement: Supplementary material 6 — Trees resulting from species delimitation under the bPTP model for COI haplotypes of S. prolifera specimens [file zookeys-1264-159_article-170411__-s006.tif]

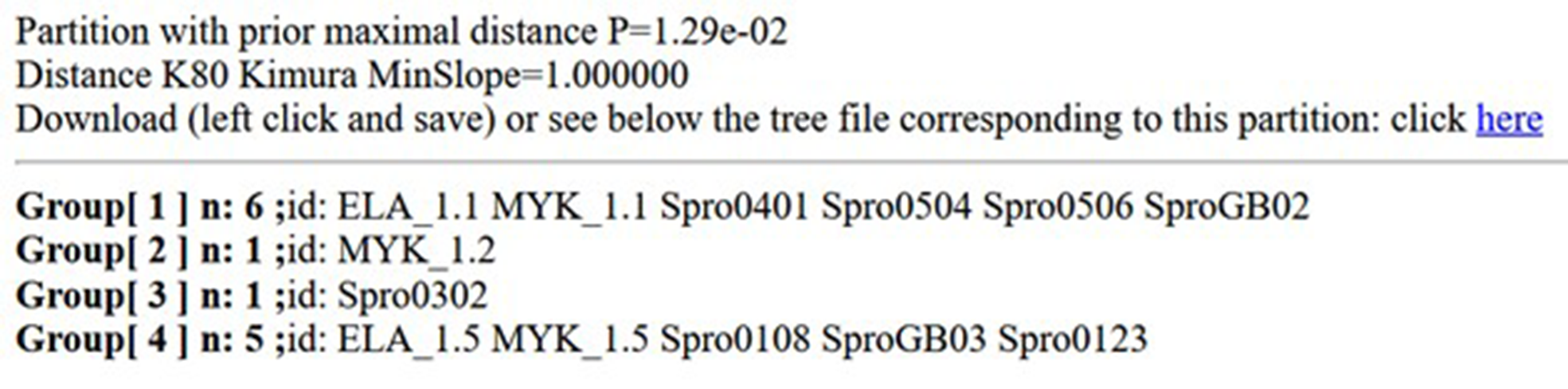

Supplement: Supplementary material 8 — Species delimitation results for ABGD model for the 16S haplotypes from S. prolifera individuals [file zookeys-1264-159_article-170411__-s008.tif]

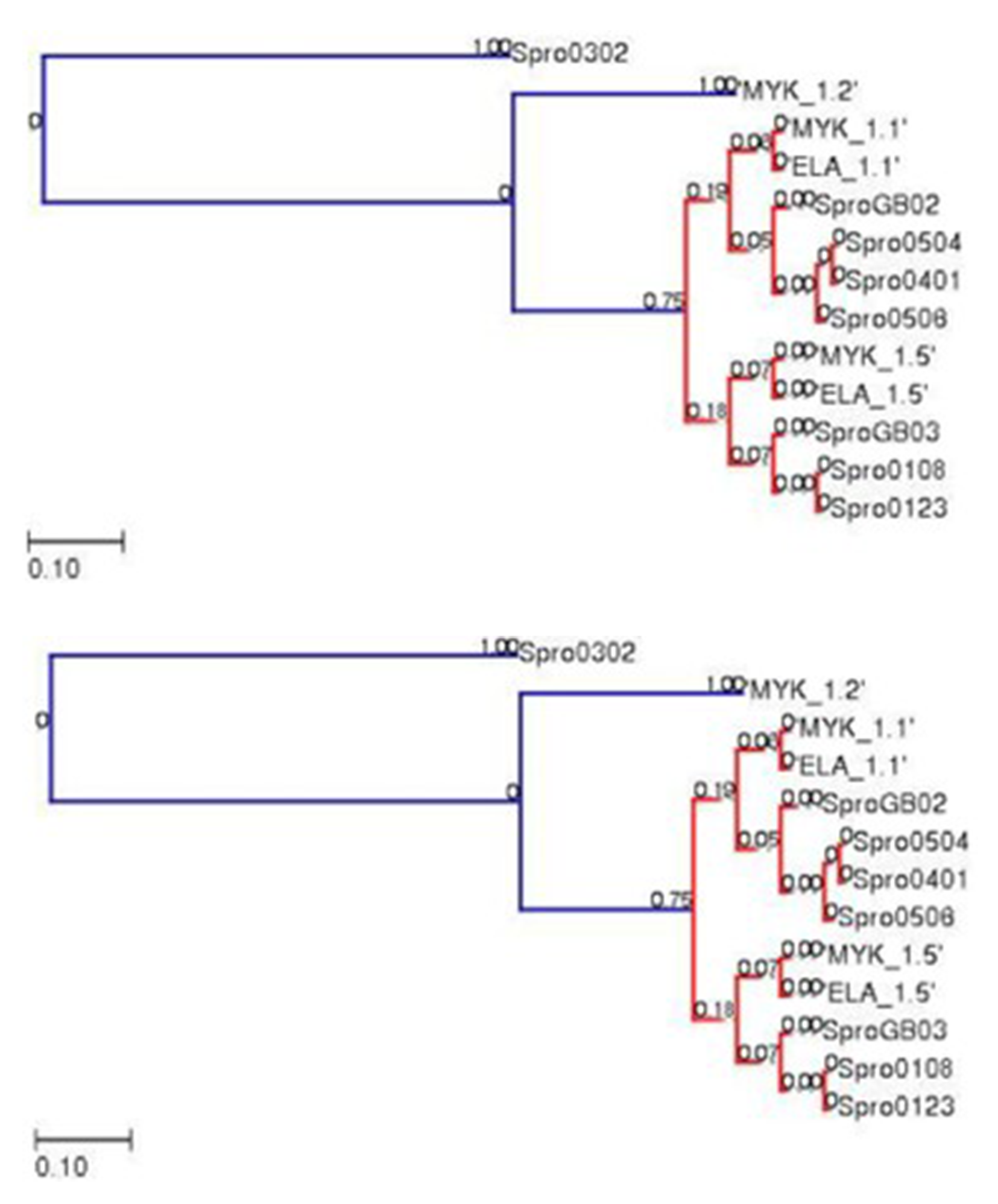

Supplement: Supplementary material 9 — Trees resulting from species delimitation under the bPTP model for 16S haplotypes of S. prolifera specimens [file zookeys-1264-159_article-170411__-s009.tif]
